# Immune mechanisms in fibrotic pulmonary sarcoidosis

**DOI:** 10.1183/16000617.0178-2022

**Published:** 2022-12-21

**Authors:** Praveen Weeratunga, David R. Moller, Ling-Pei Ho

**Affiliations:** 1 Oxford Sarcoidosis Clinic, Oxford Interstitial Lung Disease Service, Oxford, UK; 2 MRC Human Immunology Unit, University of Oxford, Oxford, UK; 3 Department of Medicine, Johns Hopkins University, Baltimore, MD, USA

## Abstract

Sarcoidosis is an immune-mediated disorder. Its immunopathology has been steadily mapped out over the past few decades. Despite this, the underpinning mechanisms for progressive fibrotic sarcoidosis is an almost uncharted area. Consequently, there has been little change in the clinical management of fibrotic sarcoidosis over the decades and an unfocused search for new therapeutics. In this review, we provide a comprehensive examination of the relevant immune findings in fibrotic and/or progressive pulmonary sarcoidosis and propose a unifying mechanism for the pathobiology of fibrosis in sarcoidosis.

## Abstract 2

**This review provides a comprehensive examination of relevant immune findings in fibrotic sarcoidosis and draws on these to propose a unifying mechanism for the pathobiology of disease.** https://bit.ly/3TR4SaB

## Background

Sarcoidosis is a multi-system immune-mediated, granulomatous disease characterised by the presence of non-necrotising epithelioid granuloma accompanied by varying degrees of lymphocytic inflammation. Activated CD4 T-cells with T-helper (Th) 1 and Th17/Th17.1 bias [1–3] drive granuloma formation, while abnormalities in regulatory T-cells [4] and invariant natural killer T-cells (iNKTs) [5, 6] may also contribute to this axis of pathogenesis by weakening the control of proliferation and activity of effector T-cells. A major recent mechanistic advance is the finding that uninhibited mammalian target of rapamycin (mTOR) signalling, possibly in lung macrophages, could be central to the unbridled formation of granulomatous aggregates [7]. mTOR complex 1 (mTORC1) senses and integrates microenvironmental signals to regulate the metabolism and proliferation of many cells, including Th1, Th17 and T follicular helper cells. Loss of control of this sensing pathway and an abnormal interaction between mTOR and autophagy have been proposed to impair antigen clearance and promote the progression of granuloma and disease chronicity [8].

**FIGURE 1:**
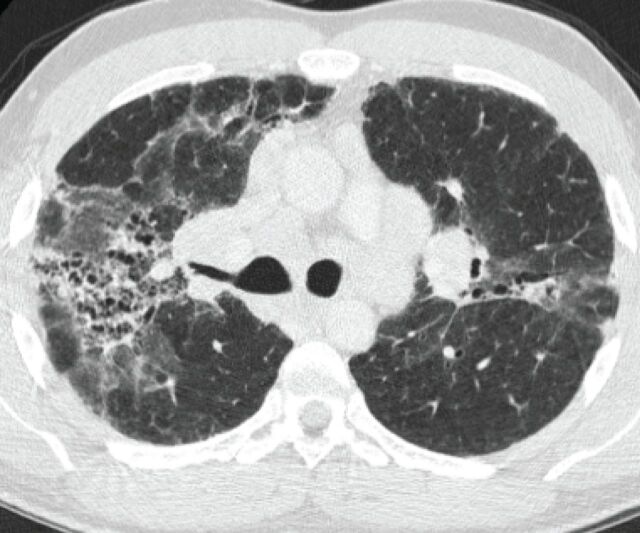
High-resolution computed tomographic section of a patient with active fibrotic sarcoidosis, with typical appearance of peri-hilar fibrocystic changes and accompanying ground-glass changes. Several patterns of fibrosis can be found but accompanying ground-glass changes could signify concomitant presence of cellular infiltrate or fine fibrosis.

The general management of pulmonary disease in sarcoidosis is relatively well established [9–11] and most patients do well. Treatment includes corticosteroids as the first-line therapy for progressive disease, with methotrexate and azathioprine as corticosteroid-sparing drugs or second-line therapy [12]. Spontaneous remission of disease occurs in approximately 50% of patients without treatment [9]. For the rest, immunosuppressants control and eventually terminate disease activity in most patients. However, a small group of patients continue to have persistent, active disease that is difficult to control. In the lungs, this can lead to progressive fibrocavitary disease (around 20% of patients) (figure 1) [13]. These patients can develop complications such as pulmonary hypertension, recurrent infection and development of mycetoma in fibrocystic areas. At present, there are no successful therapeutic drugs for these patients. Most patients with fibrotic sarcoidosis are treated with corticosteroids and multiple immunosuppressants, which can increase susceptibility to infections, potentially stimulating further fibrosis [14]. For pulmonary sarcoidosis, this is the subset of patients with the greatest need for better management strategies and new therapeutic agents. Exciting outcomes from the INBUILD study on progressive fibrotic lung disease suggest that patients with fibrotic sarcoidosis could benefit from nintedanib, a triple kinase inhibitor currently used in idiopathic pulmonary fibrosis (IPF) [15]. However, fewer than 11% of the patients in the study had sarcoidosis and they were grouped with “other fibrotic interstitial lung disease”. Therefore, it is still not clear how patients with fibrotic sarcoidosis will respond to this drug in the real world and more research is needed to find new drugs and management approaches.

Fibrotic pulmonary sarcoidosis carries a significant mortality burden. A retrospective French cohort study of patients (n=142) with stage 4 Scadding radiograph changes (fibrosis) showed that these patients had a mortality rate of 16% at 10 years from the point of diagnosis [16]. Extent and progression of fibrosis and the development of secondary complications were important determinants of survival. In a case control study of 251 patients with pulmonary sarcoidosis, the presence of 20% or more fibrosis on thoracic computed tomography (CT) were among three features with a clear prognostic predictive value [17]. Sarcoidosis patients in the US's United Network for Organ Sharing transplant database with end-stage fibrotic pulmonary sarcoidosis had a mortality rate of 28.1% in a 6-year period [18]. These figures are comparable to mortality rates for IPF patients within the same end-stage fibrotic cohort.

A significant problem with the management of fibrotic sarcoidosis is that it is not possible to predict which patients proceed to fibrosis. Indeed, there are no large-scale prospective studies that examine the incidence and risk factors for developing fibrosis in pulmonary sarcoidosis. Many patients with fibrotic sarcoidosis can have very few symptoms during the active fibrotic stage, preventing them from seeking medical attention and possibly delaying their presentation to a stage that is less amenable to anti-inflammatory treatments. These factors, coupled with a poor understanding of the causes of progression of granulomatous inflammation to fibrosis have hampered development of robust management strategies and identification of patients for clinical trials.

**FIGURE 2:**
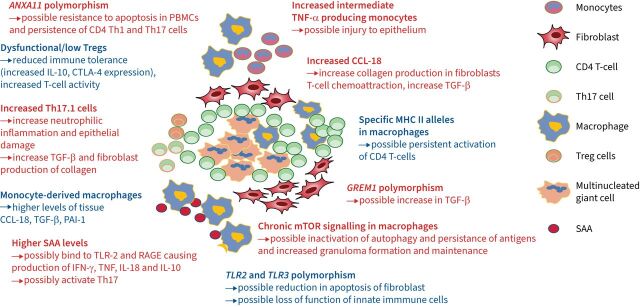
Current key immune drivers of fibrosis in sarcoidosis. Red text highlights those cells/pathways with evidence from fibrotic sarcoidosis. Blue text indicates a likely contribution derived from evidence in chronic/progressive/active sarcoidosis. A heavy T-lymphocyte involvement is likely in active fibrotic sarcoidosis, possibly with secretion of interleukin (IL)-17 from activated T-lymphocytes. C-C motif chemokine ligand 18 (CCL-18) secreted by macrophages could promote accumulation of T-lymphocytes and secretion of collagen by macrophages. *GREM1* polymorphisms which affect transforming growth factor-β (TGF-β) production in T-cells or macrophages is a potential contender. Strongest evidence probably lies with chronically increased mammalian target of rapamycin (mTOR) signalling and reduced autophagy in macrophages which allows persistence of antigen, chronically activated macrophages and perseverance of granuloma and disease activity. It is currently unclear which of these pathways are critical, and how many of these factors have to co-exist to result in progressive fibrotic disease in sarcoidosis. CTLA: cytotoxic T-lymphocyte-associated antigen; IFN: interferon; MHC: major histocompatibility complex; PAI-1: plasminogen activator inhibitor 1; PBMC: peripheral blood mononuclear cell; RAGE: receptor for advanced glycation end-products; SAA: serum amyloid antigen; Th: T-helper; TLR: Toll-like receptor; TNF: tumour necrosis factor; Treg: regulatory T-cell.

In this review, we draw together and discuss the findings and potential immune mechanisms underlying fibrosis in sarcoidosis. Specifically, we are interested in why some patients progress to fibrosis while others do not and the factors contributing to progression to fibrosis, rather than susceptibility to the disease itself. As very few studies have deliberately studied fibrotic sarcoidosis, we also gleaned information from publications that include chronic active sarcoidosis or progressive radiological disease and state these qualifications against the relevant reports. We considered chronic progressive active sarcoidosis as a prerequisite for fibrosis, but note that the majority of such patients do not develop fibrosis if treated and many remit spontaneously, the latter usually within 6 months. In some studies, late-stage disease was compared to Lofgren's syndrome (the acute and usually nonprogressive form of disease) and these are highlighted. The definition of disease activity and disease progression varies between studies and these are emphasised where appropriate. In all cases, Scadding chest radiograph stages are defined as follows: stage 1 – no lung parenchymal abnormalities visible, mediastinal and/or bilateral hilar lymphadenopathy only; stage 2 – presence of lung parenchymal abnormalities and bilateral hilar lymphadenopathy; stage 3 – presence of lung parenchymal abnormalities but no bilateral hilar lymphadenopathy; and stage 4 – chest radiograph presence of lung fibrosis with variable lung parenchymal abnormalities and lymphadenopathy [19]. We start with genetic variants that might predispose sarcoidosis patients to fibrotic pulmonary sarcoidosis, then transcriptomic findings and finally cellular profiling studies. In several instances, the relevant mechanisms or findings are compared between IPF and fibrotic or progressive sarcoidosis. These are collated in table 1. We then evaluate the rationale and relevance of the studies and draw an overall interpretation from the findings (figure 2).

**TABLE 1:** Summary of immune findings in fibrotic or progressive sarcoidosis compared to idiopathic pulmonary fibrosis (IPF), as documented in the text

	Fibrotic sarcoidosis or progressive sarcoidosis	IPF
***TLR3* polymorphism**	Polymorphism Leu412Phe (rs3775291) associated with persistent disease at 2 years [39]	Polymorphism Leu412Phe (rs3775291) associated with disease progression and mortality [38]
**Transcriptome of lung tissue**	Similar to hypersensitivity pneumonitis [46]	Different compared to hypersensitivity pneumonitis [46]
**Type I IFN signature**	Three IFN-stimulated genes upregulated in blood immune cells in more severe compared with milder disease [48]	Enrichment of type I IFN signature in monocytes [50] and a subset of macrophages [51] associated with amount of fibrosis in IPF
**Monocyte levels**	Increased levels in sarcoidosis patients with progressive disease (2-year follow-up) [76]	Increased levels correlate with higher mortality [75] and amount of fibrosis on CT [50]
**SAA levels**	Higher serum levels in fibrotic *versus* nonfibrotic sarcoidosis [64]; amount of SAA staining in lung tissue correlated with fibrosis [63]	Higher serum levels compared to healthy control [64, 65]
**CCL-18**	Unstimulated production of CCL-18 in BAL cells increased progressively comparing Scadding stages 1–4 [72]	Serum levels linked to mortality [71] and unstimulated production of CCL-18 in BAL cells higher than control and fibrotic sarcoidosis [72]
**CD163^+^ macrophage presence in lung tissue**	Increased levels in lung and lymph nodes, linked to progression [80]	Increased levels in lungs [81, 82]
**Th17**	Th17 cells and IL-17 expression has been shown to be both raised and reduced in sarcoidosis lung, BAL and granuloma [1, 85, 91–99]. PD-1^+^ Th17 cells increased in blood of fibrotic/progressive sarcoidosis patients [100]	Th17 cells reduced in blood of IPF patients [142]. PD-1^+^ Th17 increased in blood of IPF [100]
**Tregs**	Lower numbers in BAL [4, 125] and dysfunction in active sarcoidosis [4, 126]	Tregs function impaired in IPF [120]
**Fibroblastic foci**	No difference in transcriptome of fibroblastic foci comparing sarcoidosis and IPF [133]	No difference in transcriptome of fibroblastic foci comparing sarcoidosis and IPF [133]

BAL: bronchoalveolar lavage; CCL: C-C motif chemokine ligand; CT: computed tomography; PD: programmed cell death; IFN: interferon; IL: interleukin; SAA: serum amyloid antigen; Th: T-helper; TLR: Toll-like receptor; Treg: regulatory T-cell.

## Insights from genetic susceptibility studies

### Transforming growth factor β (TGF-β)

TGF-β is a major regulator of both physiological and pathological repair (fibrogenic) processes [20]. Inhibition of TGF-β signalling ameliorates fibrosis [21]. It is produced by different cells including macrophages, T-lymphocytes, bronchial epithelial cells and type II alveolar epithelial cells, and can promote direct differentiation of fibroblasts into collagen-secreting myofibroblasts [22]. Their contribution to fibrosis is complex and the cellular origin may influence its effects. For example, *TGFB1* isoforms from macrophages promote fibrosis, while those originating from regulatory T-cells (Tregs) have an opposite effect [23]. In addition, TGF-β has anti-inflammatory effects and patients with *TGFB* polymorphisms may well be those for whom disease does not progress to fibrosis due to the quenching of inflammation and halting of chronic inflammation, repair and fibrosis.

In a study on sarcoidosis, *TGFB* genetic polymorphisms were examined in patients with acute or self-remitting pulmonary sarcoidosis (n=50), chronic disease with fibrosis (n=24) and those without fibrosis (n=34) compared to those with Lofgren syndrome (n=46) and a healthy control group (n=315). Disease categories were allocated after a 4-year follow-up period. The *TGFB3* 4875 A (OR 7.9), *TGFB3* 17369 C (OR 5.1) and *TGFB2* 59941 G alleles (OR 2.9) were over-represented in chronic fibrotic patients compared to those with acute/self-remitting and nonfibrotic chronic sarcoidosis. Interestingly, *TGFB1* gene polymorphisms were not associated with fibrosis [24]. Further studies evaluating the role of polymorphisms in the *TGFB* pathway in 32 patients with fibrosis (chest radiograph Scadding stage 4) also demonstrated an association with *TGFB3* with a mutated C allele, but there was a lower *TGFB2* G-allele mutation frequency [25]. It is not clear if these polymorphisms have functional consequences. To complement this, serum and lung levels of TGF-β in sarcoidosis have been examined over several decades, with conflicting results. One study found raised TGF-β1 levels in bronchoalveolar lavage (BAL) fluid from sarcoidosis patients with altered lung function (not specifically with fibrosis) compared to those with normal lung function [26]. However, subsequent studies demonstrated that serum TGF-β1 levels were significantly higher in patients with Scadding stage 0–1 sarcoidosis (n=18) compared to normal healthy control patients and those with stage 4 disease (n=13) [27]. Similar observations were made when examining samples from 16 sarcoidosis patients, obtained by exhaled breath condensate, where unbound TGF-β1 levels did not correlate with radiological stage (albeit that there were only two Scadding stage 4 patients) [28]. As it is widely acknowledged that most bioactive and unbound TGF-β acts within the microenvironment in which it is released, it is likely that the lung findings are more relevant than the serum.

Examining the possible involvement of *TGFB* from another angle, *GREM1* polymorphisms were examined by several investigators in fibrotic sarcoidosis patients. *GREM1* encodes Gremlin, which inhibits bone morphogenic proteins (BMPs), a group of growth factors from the TGF-β superfamily that counteract the action of TGF-β. The balance between TGF-β and BMP signalling is thought to be an important determinant of a fibrotic response [29, 30]. Significant differences were found in the *GREM1* polymorphisms between sarcoidosis patients without chest radiography abnormalities (n=116) compared to patients with fibrosis on chest radiography (n=59). The most significant association was with *GREM1* rs1919364. Carriers of the *GREM1* CC genotype at position rs1919364 were 6.4 times more likely to develop lung fibrosis [31].

### Toll-like receptors (TLRs)

Variants of the TLR genes have also been implicated in chronic and progressive sarcoidosis. TLRs are a group of pattern-recognition receptors that are integral to the ability of the innate immune system to recognise, activate and regulate the interaction between innate immune cells and microbial and other antigens [32]. Different TLRs are found in different parts of cells and they recognise different pathogen-associated molecular patterns (PAMPs), *e.g.* TLR4 in the plasma membrane detects lipopolysaccharide (LPS), TLR5 detects flagellin and TRL1, 2 and 6 recognise bacterial lipoproteins. Those in the endosome, such as TLR3 and TLR8, detect nucleic acids (double-stranded and single-stranded RNA, respectively). Of these, TLR2 has been most frequently implicated in the pathogenesis of sarcoidosis [33–35]. Heron *et al*. [31] studied the prevalence of *TLR2* polymorphisms in chronic sarcoidosis (using radiographic progression at 4 years) and found variable results in its link to chronicity [36]. However, in a later study, the same investigators classified sarcoidosis patients as those with or who had developed pulmonary fibrosis within 4 years compared to those with self-limiting or Lofgren's syndrome and found that a haplotype of single nucleotide polymorphism (SNP) variants affecting *TLR1*, *TLR6* and *TLR10* genes (which can act as co-receptors with TLR2) were absent in the fibrotic group [37]. Furthermore, the allele frequencies for rs1109695, rs7658893 (*TLR10*) and rs5743604 (*TLR1*) in chronic fibrotic patients differed significantly from those of healthy controls. However, there were no differences in the above allele frequencies between sarcoidosis patients with pulmonary fibrosis and those without fibrosis.

In another study, the *TLR3* polymorphism Leu412Phe (rs3775291), which was associated with accelerated disease progression and elevated mortality risk in IPF [38], was evaluated in patients with sarcoidosis. In this study there was a significant association between this *TLR3* polymorphism and persistent clinical disease in two cohorts of Irish and American Caucasians with pulmonary sarcoidosis. “Persistent disease” was determined at 2 years and defined as 1) patients who were at Scadding stage 2 or 3 on chest radiograph with associated abnormal pulmonary function parameters (forced vital capacity (FVC) and/or total lung capacity and or transfer factor <80% of predicted values), 2) patients with Scadding stage 4 chest radiograph, or 3) patients who were prescribed corticosteroids. In a very small sub-study (n=3 patients), activation of *TLR3* in primary lung fibroblasts from 412 F-homozygous pulmonary sarcoidosis patients resulted in reduced *IFNB* and *TLR3* expression, and reduced apoptosis and dysregulated fibroproliferative responses compared with *TLR3* wild-type patients [39]. The functional studies are probably too small to draw a conclusion. However, the correlation of *TLR3* polymorphism with persistent disease in sarcoidosis (some of which with fibrosis) and highly progressive IPF suggests that it is possible that the TLR3 signalling pathway anomalies are involved in susceptibility to fibrotic pulmonary sarcoidosis, either *via* loss of function in innate immune cells like macrophages or reduced apoptosis in lung fibroblasts [38].

### Annexin A11

Annexin A11 is encoded by *ANXA11* and was the first gene linked to disease susceptibility in sarcoidosis using the genome-wide association studies approach [40, 41]. Polymorphism in the C allele of SNP rs1049550 was associated with a significantly increased risk of sarcoidosis in a German population [40]; a finding that was replicated in two other European populations [42, 43]. Annexin A11 is a cytosolic, calcium-dependent protein with diverse functions. *ANXA11* is most highly expressed in whole blood cells, particularly B lymphocytes, monocytes and some subsets of immature myeloid cells. Peripheral blood mononuclear cells (PBMCs) isolated from sarcoidosis patients who carried the *ANXA11* R230C SNP were more resistant to apoptosis than the wild genotype, although this was a small study [44]. In an African American cohort followed up for at least 2 years, a minor allele was observed to associate with radiographically persistent disease (stage 4 Scadding) [41]. This could be explained by the persistence of immune cells such as CD4 Th1 or Th17 (due to their greater resistance to apoptosis conferred by the polymorphism), resulting in continued inflammation and persistence of granuloma.

In general, studies on polymorphisms in fibrotic sarcoidosis are small and few replicative cohorts were examined. As such, it is difficult to draw definitive conclusions from them; apart perhaps from the *GREM1* studies [45] and the *ANXA11* findings [40–43]. These abnormalities suggest that a loss or gain in the ability of the innate immune system to sense pathogens and the persistence of the immune response due to resistance to apoptosis could contribute to disease progression and possibly fibrosis in sarcoidosis.

## Insights from transcriptomic studies

Transcriptomic studies offer the next layer of insight into possible causes of fibrotic sarcoidosis by linking gene expression in cells or tissue with possible functional abnormalities.

In one of the first transcriptomic studies in lung tissue from sarcoidosis patients, Lockstone *et al.* [46], compared the gene expression profile of lung tissue from patients with self-limiting disease to those with progressive and/or fibrotic disease [47]. In the progressive/fibrotic group, patients had fibrosis and nodules on CT scans, abnormal lung function, and persistent or progressive chest radiograph changes over 2 years. Compared to the group with self-limiting, nonfibrotic disease, the transcriptomes of lung tissue from these patients were enriched with gene sets involved in “leukocyte activation and differentiation”, “response to stimuli” and “cytokine production”. Other major processes that were found to be upregulated in the pulmonary fibrosis group included intracellular signalling (NF-κB and Janus tyrosine kinase/signal transducer and activator of transcription cascades) and categories related to apoptosis, cell cycle, cell proliferation and homeostasis. These findings broadly indicate stronger immune activation and cellular activity in the fibrosis group, which, while not unexpected for sarcoidosis, underlines the importance of immune activity in the progression to fibrosis in sarcoidosis. More discerning was perhaps the finding that the differentially expressed gene list in the progressive fibrotic group was enriched with differentially expressed genes from hypersensitivity pneumonitis (HP) but not IPF [46]. This suggests that, in the lungs, an active T-lymphocyte-based immune response (as was found in the HP gene set) is likely to be key to progressive fibrotic pulmonary sarcoidosis, distinct from the fibroproliferative mechanisms in IPF.

An important study from Koth *et al.* [48] followed, which examined the difference in the gene expression profile of blood cells between sarcoidosis patients and healthy controls. Although no characterisation of chronicity, disease progression or fibrosis was performed, the authors examined genes that discriminated disease severity according to lung function (forced expiratory volume in 1s and/or FVC< 80% predicted). Among the 10 genes that were differentially expressed, three were interferon (IFN)-inducible genes or encoded for mediators downstream of the IFN signalling pathway. The expression of one of these genes, IFN regulatory factor 1 (*IRF1*), a transcription factor stimulated by type I and II (but not the anti-inflammatory type III) IFNs, was significantly different between sarcoidosis patients with reduced lung function compared to those with normal lung function [49]. *IRF1* is a critical transcription point in the IFN signalling pathway and its engagement stimulates the transcription of a specific set of pro-inflammatory chemokines which recruit both innate and adaptive immune cells to the site of activation [49]. This is an interesting finding as it matches the finding of type I IFN enrichment in IPF both in monocytes [50] and in a subset of macrophages (*SPP1*^+^ macrophages) [51]. Both of these findings have been associated with the extent of fibrosis in the lungs of IPF patients.

Koth *et al.* [48] also questioned if genes identified in lung tissue from progressive fibrotic sarcoidosis patients (derived from Lockstone *et al.* [46] and another study (Ohio study)) were also differentially expressed in peripheral blood cells. Among the genes that were concomitantly induced in the lung and blood in sarcoidosis was a critical transcriptional regulator in the type I and II IFN signalling pathway – *STAT1 –* and sets of genes related to IFN signalling pathways. The type II IFN (IFN-γ) signalling data are consistent with it playing a significant role in Th1-type inflammation in sarcoidosis, which is not unexpected. However, the involvement of type I IFN genes is interesting and corroborates the findings mentioned above [50] that linked monocytes primed for type I IFN signalling with pathogenesis of IPF [50]. This raises the possibility of a pro-fibrotic role for type 1 IFN-primed monocytes in fibrotic sarcoidosis [50]. Of note, treatment with IFN-α and IFN-β has been associated with new-onset or recurrent sarcoidosis [52, 53], providing further support for the pathogenic role of type 1 IFN in sarcoidosis.

The next significant advance came from Vukmirovic *et al*. [54], who performed bulk RNA sequencing of BAL cells from 209 patients as part of a large comparative study in the United States (Genomic Research in Alpha-1 Antitrypsin Deficiency and Sarcoidosis (GRADS) study). When the gene expression profiles were analysed without an *a priori* hypothesis, four gene modules were identified that corresponded to four clinical groups, one of which being chronic sarcoidosis. Weighted gene co-expression network analysis, a bioinformatics application for exploring the relationships between groups of gene sets (representing a module) that change together with clinical features or types, was able to match modules of genes to these four “endotypes”. For the chronic sarcoidosis endotype, a 51-gene module was identified, although the identity of the driver genes was not published. However, in the supervised analysis comparing patients with Scadding stage 1 chest radiographs (n=54) to stage 4 (n=33), gene sets that correlated with Scadding progression showed enrichment with interleukin (IL)-6, IL-8 signalling, stem cell reperfusion and IL-1 signalling pathways. In the fibrotic (stage 4) group, cell cycle, signal transduction in mTORC1 and high shear stress induced platelet activation gene sets were found to be upregulated. In terms of specific genes, there was increasing expression of *PLA2G7*, *ID1*, *LGMN*, *SLC40A1* and *CCL2* and reducing expression of *PDLIM1* and *AOC3* with increasing Scadding stage. Patients with reticular abnormality on CT scans showed upregulation of fibrosis-associated genes such as *TGFBR1*, *COL3A1*, *TLR3*, *ID1*, *TCF4*, *IGFBP6*, *PLA2G7*, *FADS1*, *ARGHAP12* and *MMP10. TLR3* is the notable finding here given previous studies, as discussed above [38, 39]. A recent study described *PLA2G7* as a pro-inflammatory pathway (potentially *via* NLRP3 inflammasome activation) that can be modulated by calorific restriction, pointing both to a potential mechanism of action for *PLA2G7* in persistent disease and fibrosis, and also an accessible lifestyle-modifying treatment [55].

The most relevant findings here are mTORC1 upregulation in the group of patients with fibrotic CT scans and, possibly, the involvement of monocytes or monocyte-derived macrophages (given the monocyte-specific chemokine, C-C motif chemokine ligand 2 (CCL-2)) in the progression of Scadding stage 1 to stage 4 (fibrosis). Uninhibited mTOR signalling in macrophages has already been shown to be a feature of excessive granuloma formation [7], making this a particularly interesting pathogenic pathway for progressive fibrotic sarcoidosis. In addition to the persistence of antigens *via* inactivation of Unc-51-like autophagy-activating kinase and autophagosome formation [56], a small study has shown that the sarcoidosis granuloma had greater phago-lysosomal activity (where mTORC1 activity occurs) compared to tuberculosis [57]. These gene expression data, in lung cells, from a large cohort and identified without an *a priori* hypothesis, are powerful considerations for the interpretation of other cellular findings, as discussed below.

One caveat to these data is that they were from bulk RNA sequencing of all BAL cells and, therefore, do not pinpoint the cellular origins of the differentially expressed genes. However, newer studies using single-cell RNA sequencing should be able to address this. Single-cell RNA sequencing studies are currently limited to a few datasets in peripheral blood [58] and BAL [59]. Although none of these included patients with fibrotic disease, Liao *et al.* [59] showed, in the first published single-cell transcriptomic data on BAL cells from sarcoidosis patients, that those with progressive compared to nonprogressive sarcoidosis (n=2 each) had increased expression of five genes in their macrophages, three of which were major histocompatibility complex (MHC) II alleles, namely, human leukocyte antigen (HLA)-B, HLA-DQA2 and HLA-DRB5. Susceptibility to sarcoidosis is influenced by many genes, but the strongest associations have been described for the HLA region [60]. Many have argued that the strong linkage disequilibrium in this region indicates that HLA association links to other genes, *e.g.* tumour necrosis factor-α (TNF-)α. However, here, in single-cell transcriptomic analysis, the finding that macrophages of those with progressive disease have a higher expression of certain MHC II allele points more to their specific role, possibly in persistent activation of CD4 T-cells and maintenance of granuloma. In a re-analysis of five transcriptomic datasets, including those from Liao *et al.* [59] and Lockstone *et al*. [46], the same authors show that “programmed cell death (PD)-1/PD-L1 cancer immune therapy” and “neuro-inflammation” gene sets were the most consistently upregulated sets, comparing progressive to nonprogressive sarcoidosis (five datasets from five studies). In addition, IL-17 signalling pathways were enriched in lungs but not blood in several of these datasets [61]. Both IL-17-producing and PD-1/PD-L1-expressing T-cells are discussed later but it is important to note that these are small studies (albeit single-cell analyses) and not in fibrotic sarcoidosis.

## Insights from circulating soluble mediators and proteins

In terms of measured protein in blood and lungs, we highlight three due to the strength of evidence and rationale.

### Serum amyloid antigen (SAA)

SAAs are acute phase-response proteins. They are cytokine-like proteins involved in cell–cell communication in inflammatory, immune, neoplastic and protective pathways [62]. SAA can act as a chemical signal, working *via* receptors such as TLR2 and the receptor for advanced glycation end-products, and aggregates of SAA have been found in granuloma. SAA has been shown to activate NF-κB in TLR2 – expressing human cell lines and regulating experimental Th1-mediated granulomatous inflammation through IFN-γ, TNF and IL-10. Furthermore, SAA has the potential to stimulate production of TNF, IL-10 and IL-18 in lung cells from patients with sarcoidosis [63].

Chen *et al*. [63] demonstrated that, in sarcoidosis lung samples with fibrosis (n=6), the extent of SAA staining positively correlated with the degree of collagen deposition. Following on from these data, Beijer *et al*. [64] measured SAA levels in serum from patients with sarcoidosis (n=215), hypersensitivity pneumonitis (n=30), (eosinophilic) granulomatosis with polyangiitis (n=11) and IPF (n=68). Patients with Scadding stage 4 had the highest serum SAA levels and these were significantly increased compared to those without fibrosis. SAA levels also correlated negatively with diffusion capacity for carbon monoxide in patients with sarcoidosis. Both this and the paper by Vietri *et al*. [65] on IPF showed that SAA levels were higher in the serum of IPF patients compared to healthy controls, linking SAA levels to fibrosis in general rather than sarcoidosis or fibrotic sarcoidosis *per se*.

It is not clear whether SAA drives progression or if it is a by-product of inflammation and fibrosis. SAA can aid antigen clearance and may be upregulated within fibrotic niches to perform antifibrogenic function *via* polarisation of macrophage differentiation to M2-like, anti-inflammatory macrophages [66]. However, SAA production by intestinal epithelium has been shown to induce Th17 responses, chronic inflammation and subsequent fibrosis [67], which supports a fibrogenic role for this protein.

### CCL-18

CCL-18 is a cytokine secreted by the myeloid lineage cells – macrophages, dendritic cells and keratinocytes. It can induce fibrogenesis through a multitude of mechanisms including collagen production in human lung fibroblasts [68]. In macrophages, CCL-18 is induced in M2-like or alternatively activated macrophages by IL-4 and IL-13. These macrophages are thought to be involved in repair and fibrosis [69]. In CCL-18 overexpressing mice, CCL-18 appeared to selectively promote perivascular and peribronchial infiltration of T-cells, with a corresponding accumulation of collagen and the presence of active TGF-β1 protein [70]. CCL-18 has been implicated in the pathogenesis of several fibrotic lung diseases and has been linked to mortality in patients with IPF [71]. In another study, which also included hypersensitivity pneumonitis patients (n=69) and healthy volunteers (n=22), spontaneous production of CCL-18 levels from BAL cells increased progressively when comparing Scadding stages 1–4 and was highest in the supernatant of BAL cells from IPF lungs [72]. CCL-18 levels also correlated with an increase in the pulmonary fibrotic burden as estimated by chest radiograph. These findings are consistent with observations by Pechkovsky *et al.* [73], who also demonstrated a progressive increase in CCL-18 in BAL fluid from Scadding stages 1 to 4.

### IL-5, IL-7 and granulocyte–macrophage colony-stimulating factor (GM-CSF)

In a comprehensive review of the circulating cytokines and chemokines in sarcoidosis patients, which included a comparison between fibrotic (n=19) and nonfibrotic (n=21) sarcoidosis, IL-7 was one of three cytokines (the other two were IL-5 and GM-CSF) that were significantly different between the fibrotic and controls but not between fibrotic and nonfibrotic sarcoidosis [74]. The most significant difference was found for IL-5, which is discussed later.

## Insights from cellular immune profiling

### Monocytes

The role of monocytes in the pathogenesis of lung fibrosis has gained traction in recent years, with increased levels in blood observed in IPF patients, and correlation with amount of fibrosis and possibly with outcome and progression [50, 75]. An excellent study by Lepzien *et al*. [76] reported findings from functional, phenotypic and transcriptomic analyses of BAL and blood for monocytes, macrophages and dendritic cells in 108 patients with sarcoidosis and 30 healthy controls over a 2-year follow-up period. Patients with chronic progressive disease (defined as deterioration in symptoms and increase in chest radiological abnormalities compared to previous assessment) showed higher frequencies of circulating intermediate monocytes (CD14^hi^CD16^hi^; nominally acknowledged as the inflammatory monocyte subset) at the time of diagnosis. In BAL cells, intermediate monocytes that were producing TNF-α without stimulation were the only correlate of disease progression. There were only two stage 4 Scadding patients, so these correlates only applied to disease activity and progression, and not fibrosis.

### Macrophages

Macrophages are an integral part of granuloma in sarcoidosis. For at least a decade, an *in vitro* model of macrophage differentiation suggested that there were two polar subsets of macrophages. Macrophages that mainly secreted pro-inflammatory cytokines were called classically activated macrophages (M1), which can be activated either by IFN-γ or LPS, and macrophages that attenuated inflammation and encouraged wound repair were referred to as alternatively activated macrophages (M2). The latter were activated by IL-4 or IL-13 [77]. Fibrotic remodelling is nominally thought to be mediated by alternatively activated macrophages, possibly through production of TGF-β and other signalling molecules such as CCL-18. However, this *in vitro* model of macrophage polarisation is unlikely to be strictly valid *in vivo*, and there is less evidence of this polarisation in humans. It is also now recognised that macrophage plasticity dictates the presence of several intermediate states of macrophages [78]. For example, we have shown by analysis of single-cell transcriptomic data from human IPF lungs that at least five states of lung macrophage exist, with expression of different inflammatory and pro-repair genes [50]. Chakarov *et al.* [79] have also identified at least two subsets of interstitial tissue macrophages in murine lungs, one with anti-inflammatory effects.

These caveats aside, there is evidence for M2-like macrophages in fibrotic sarcoidosis. Shamaei *et al.* [80] examined tissue samples from mediastinal lymph nodes and transbronchial lung biopsy in 10 patients with sarcoidosis, of which two patients had evidence of fibrosis (Scadding stage 4). A notable increase in CD163^+^ M2 macrophage populations and giant cells in the lung and lymph node sections was reported with a significant association with radiological progression. However, it is unlikely that CD163^+^ macrophage presence is specific to fibrotic sarcoidosis as it has also been shown in IPF lungs [81, 82]. A much larger study of 102 sarcoidosis patients showed progressively increased levels of CCL-18 and CCL-17 (a CD4 T-cell chemoattractant), but not TNF-α, in BAL of Scadding stage 1–4 patients (n=32/35/23/12, respectively). These mediators were measured due to their purported association with M1(TNF-α) and M2 (CCL-18) macrophages, but, in their own right, these findings are interesting as they suggest that TNF-α may not be associated with fibrosis in sarcoidosis, despite it being a correlate with disease activity and progression [73].

More recent data from Jeny *et al*. [83] suggest that macrophages differentiated from monocytes isolated from patients with active sarcoidosis (n=26) were more sensitive to hypoxic challenge *in vitro* than those isolated from patients with inactive sarcoidosis, producing more TNF-α, IL-1β and TGF-β1. These macrophages also secreted more PAI-1 (plasminogen activator inhibitor-1), which blocks fibrinolysis and promotes extracellular matrix accumulation in tissues. These findings link well with another study [84] that showed enrichment of hypoxia inducible factor (HIF) pathways in monocytes and macrophages from sarcoidosis patients, and downregulation of IL-17 with inhibition of HIF-1α. They implicate monocytes in active sarcoidosis and raise the possibility that monocyte-derived macrophages promote fibrosis *via* secretion of PAI-1, TGF-β1 and IL-17 within the hypoxic environment of the granuloma. However, since most active disease do not progress to fibrosis, this remains a speculation.

### Th17 and Th17.1

CD4^+^ T-cells are divided into unique subsets based on the cytokines they secrete and their distinct functional abilities. The CD4^+^ Th17 cell subset expressing the pro-inflammatory cytokine IL-17A is emerging as an important driver of fibrosis in general [85–88], possibly by promoting neutrophilic inflammation and thus epithelial damage. A recent study suggests that IL-17B produced by LPS-stimulated macrophages is critical to lung fibrosis [89]. A large genetic variant study of >19000 individuals has highlighted the Th17 pathway as a possible pathogenic factor in sarcoidosis [90]. However, Th17 cells and IL-17 expression have been shown to be both raised and reduced in sarcoidosis lung, BAL and granuloma [1, 85, 91–99]. A subset of Th17 cells, Th17.1, which also produce IFN-γ and further upregulate pro-inflammatory cytokines and confer corticosteroid resistance [93], is markedly raised in BAL from sarcoidosis patients with progressive disease [3]. Broos *et al*. [2] analysed Th17.1 cells in mediastinal lymph node cells from treatment-naive pulmonary sarcoidosis patients (n=17) and healthy controls (n=22), and PBMCs (n=34) and BAL (n=36) and followed up these patients for 2 years. Patients who did not resolve within this time had higher levels of IL-17 in their BAL compared to those whose disease resolved. None of these studies subcategorised patients into fibrotic and nonfibrotic disease.

In a study primarily on IPF patients, Celada *et al.* [100] showed that PD-1-expressing Th17 cells were increased in blood from a small number of sarcoidosis patients (n=8) compared to healthy controls but were highest in IPF patients. Interestingly, PD-1^+^ Th17 cells are the dominant producers of TGF-β and, when co-cultured with fibroblasts, induced collagen production. PD-1 blockade and STAT3 inhibition appear to reduce collagen production in these co-cultures. In another study, PD-1^+^ CD4 T-cell levels in blood were higher in patients with active sarcoidosis (defined by reduced FVC, radiographic progression or acceleration of pulmonary symptoms) compared to resolved disease [101], although IL-17 secretion by these cells was not evaluated. PD-1^+^ CD4 T-cells also demonstrated reduced proliferative capacity, and a paired BAL and blood sample comparison showed a higher level of these cells in BAL. These are important but challenging concepts as they suggest that PD-1/PD-L1 pathway activation in CD4 T-cells is associated with increased IL-17 and TGF-β production while also reducing proliferation of these cells. At the advanced end of the disease spectrum, immunohistochemical analysis of lungs of two patients with refractory fibrotic sarcoidosis showed that, in the fibrotic parts, T-cells did not express IL-23R and IL-17, and alveolar macrophages did not stain with anti-IL-17 monoclonal antibodies [1]. It is possible that an IL-17-mediated immune response is important for tipping progressive disease to fibrosis, but perhaps becomes less relevant in chronic end-stage fibrotic disease. A noteworthy point around PD-1/PD-L1 pathway activation in sarcoidosis is the report of a sarcoidosis-like reaction with the use of immune checkpoint inhibitors [102]. This has been observed for anti-cytotoxic T-lymphocyte-associated antigen (CTLA)-4, PD-1 and PD-L1 inhibitor classes of these drugs, and occurring around 3 months after commencement of treatment. Theoretically, emergence of sarcoid-like reactions is not surprising for CTLA-4 inhibitors, as CTLA-4 mediates suppression of T-cell activity. However, the appearance of sarcoidosis in PD-1 and PD-L1 inhibitor treatment is more difficult to explain given Celada *et al.*'s [100] findings above. This discordance could be due to a difference between Th17 immune activity in peripheral blood (as in the experiments in Celada *et al.* [100]) and that in sarcoidosis lungs. In cancer patients, treatment with both anti-CTLA-4 and PD-1/PD-L1 inhibitors is associated with an increase in Th17 cells [103–105], so there is not enough evidence for the use of immune checkpoint inhibitors in sarcoidosis, particularly given the likely importance of Th17 cells in progression of sarcoidosis.

### Th1/Th2 switch

The archetypal Th1 and Th17.1 cytokine, IFN-γ, is an antifibrotic cytokine, yet it is the dominant mediator of pathogenesis in sarcoidosis. It is therefore an interesting conundrum as to how fibrosis ensues within this IFN-γ-rich environment. This paradox has led many to hypothesise that a transition from Th1 to Th2 pathway activation occurs in chronic sarcoidosis, perhaps as a response to persistent inflammation [106]. Th2 cytokines (IL-4, IL-5 and IL-13) are certainly important mediators of progressive fibrosis [22, 107, 108]. Of these, IL-13 is probably the most dominant mediator of fibrotic tissue remodelling [109], inducing fibrosis by stimulating the production and activation of TGF-β and activating fibroblasts, epithelial cells and smooth muscle cells [110–112]. An interesting finding came from Patterson *et al*. [113], who analysed circulating cytokine profiles in 54 patients with biopsy-proven sarcoidosis, which included 19 patients with fibrotic pulmonary disease and 21 patients with nonfibrotic disease. Whilst not raised in fibrotic sarcoidosis (compared to healthy controls), IL-5 was significantly decreased in nonfibrotic compared to fibrotic sarcoidosis, suggesting that reduced IL-5 is protective against fibrosis. However, it is unclear how this occurs. IL-13 gene expression was significantly increased in BAL cells and PBMCs in patients with sarcoidosis, but these patients had Scadding stages 1–3 only [114]. Overall, there is no evidence of Th1/Th2 switching in fibrotic sarcoidosis. However, studies have shown that IFN-γ, while not pro-fibrotic in itself, may induce injury and inflammation which then leads to fibrosis [115, 116]. In this scenario, it is plausible that the persistent Th1-cell activity could cause low grade injury and add to the stimulus for repair and fibrosis. It is interesting to note that IFN-γ therapeutic trials in IPF did not reach their end-points [117, 118], suggesting that IFN-γ has (if any) only a minor role in fibrosis.

### Tregs

Tregs are a specialised subset of CD4^+^ T-cells that rely on the transcription factor FOXP3 for development and function. They tend to be immunosuppressive and play a critical role in the function, establishment and maintenance of immune tolerance *via* multiple mechanisms including production of inhibitory cytokines (IL-10), expression of inhibitory receptors such as CTLA-4 and the ability to deplete the inflammatory environment of pro-inflammatory cytokines such as IL-2. Furthermore, Tregs can undergo reprogramming in certain tissues and acquire the ability to suppress specific cells such as TH2 effector cells, which are potent drivers of fibrosis though production of IL-13 and IL-4 [119]. Treg cells have been shown to ameliorate fibrotic mechanisms in IPF [120], dystrophic mouse muscle [121], cardiovascular disease [122], chronic graft-*versus*-host disease-induced lupus nephritis [123] and chronic hepatitis C virus- and HIV-induced liver fibrosis [124]. Sarcoidosis patients have lower numbers of Tregs in the BAL [4, 125], and Treg dysfunction has been demonstrated in patients with active sarcoidosis [4, 126]. The ratio of Tregs/Th17 in the circulation increases in response to immune-modulating therapy and subsequently decreases during disease relapse and when immune suppressants are discontinued [127]. In an interesting study, decreased CTLA-4 expression was demonstrated in both Th17 and Tregs in patients with sarcoidosis, but only Th17 cell numbers were increased [128]. It was suggested that the TGF-β produced by Tregs differentiated naive CD4 T-cells to Th17 cells while at the same time prevented Tregs from apoptosing, thus maintaining the number of Tregs [129]. However, no studies have shown these abnormalities in fibrotic sarcoidosis specifically. Nevertheless, as there appears to be an integral relationship between Tregs and the Th17 response (the latter also linked to fibrotic sarcoidosis), and T-cell activity in general appears to have a major immune association with fibrosis, it is possible that reduced or dysfunctional Tregs could be involved in fibrosis by allowing an expansion of the relevant effector T-cells in granulomatous sites.

## Other considerations

An important clinical observation is that fibrosis in sarcoidosis typically occurs over a long period of time – 10, sometimes 20 years. Apart from rare cases of rapidly progressive fibrosis, it seems clear to us that the rate of fibrosis is much slower than that seen in IPF, connective tissue interstitial lung disease or hypersensitivity pneumonitis. This raises some interesting questions: is the pathogenesis of fibrosis in sarcoidosis different? and, intriguingly, could it be slowed down by the IFN-γ-rich milieu? Another potential contributor to this slow progression is the role of the antigen. The identity of the antigen has been elusive, but it seems from the large and definitive ACCESS study that there is no single antigen for all patients nor even an individual. Nor is it the only trigger for an immune response – host factors such as chronic stress have long been noted by clinicians to be associated with worsening of sarcoidosis and there is a well-established immunological basis for this [130]. Notwithstanding, of the many potential antigens (*e.g.* SAA, nondegradable components of *Mycobacterium tuberculosis* or *Cutibacterium acnes*, silica, pine pollen; reviewed in [9]), it is possible to speculate that those antigens that are nondegradable, *e.g.* silica dust, could be a part of a combination of factors that come together to cause chronic fibrosis.

Aside from immune cells, fibroblasts are also likely to be relevant players in the progression to chronic fibrosis in sarcoidosis. Stromal cells such as fibroblasts and pericytes have also been suggested as significant regulators of the immune system [131]. It could be hypothesised that fibroblasts in sarcoidosis patients provide a feedback signal to immune cells that contribute to perpetuation of fibrosis. No studies have tested this possibility definitively, but two small studies have provided tantalising leads. Tamura *et al.* [132] showed that fibroblast outgrowths from transbronchial biopsies of nonfibrotic sarcoidosis lungs (n=7) produced more IL-6 after stimulation with IL-1β than biopsies from IPF lungs (n=4) and cancer lungs (n=5). More recently, Kamp *et al.* [133] found that laser-dissected fibroblastic foci that were subjected to gene expression analysis of 700+ fibrosis-related genes showed no significant differences between sarcoidosis and IPF. Taken together, these data suggest that pre-fibrotic fibroblasts in sarcoidosis patients may have pro-inflammatory features which could promote injury and aberrant repair, resulting in fibroblastic activity that is similar to that seen in IPF patients.

An area that is only just starting to take off in sarcoidosis is the role of epigenetic changes in immune cells and persistence of disease. No study has yet shown a correlation between epigenetic marks and fibrotic sarcoidosis, but Yang *et al.* [134] examined the DNA methylation profile in immune cells from BAL of patients with progressive compared to remitting sarcoidosis and found subtle changes in the DNA methylation profile in a chemokine (CCL-6) and, interestingly, also in HLA-DR [134], which is worth further exploration.

## Conclusion

Transplant surgeons and pathologists noted more than a decade ago that the lungs of patients with end-stage fibrotic sarcoidosis show a distinct amount of lymphocytic infiltrates [135, 136]. Those with high levels of cellular infiltrate progressed faster to end-stage disease compared to those with a low number of immune cells (4.8 *versus* 23.3 years) [136]. In a significant proportion of patients at the transplant point (40%), granuloma still featured amid the presence of fibrosis. Although small in number, the histopathological data provides persuasive support for the importance of immune cells in driving the progression of fibrosis. They complement transbronchial biopsies from Lockstone *et al.* [46], whose sampling at an earlier stage of the disease showed that the top three gene sets that differentiated the lung tissue transcriptome of progressive/fibrotic patients and self-limiting tissue were “immune response to stimuli”, “leukocyte activation” and “cytokine production”. Diving deeper into the specific immune drivers of fibrotic sarcoidosis, in our view, the strongest data point to two broad groups of cells, Th17 cells in the adaptive immune system and CCL-18-expressing innate immune cells, and possibly macrophages *via* the recognition of PAMPS with TLR2. These mediating pathways could be facilitated by the presence of SAA in some patients, which could drive the activity of macrophages, and chronic mTORC1 signalling, which allows for the persistence of antigens in these macrophages. Although Linke *et al.*'s study [7] on mTOR signalling in the persistence of granuloma was wholly done in murine models, an association with sarcoidosis was shown *via* interrogation of the transcriptomic data from Lockstone *et al*.’s [46] progressive fibrotic *versus* self-limiting dataset in the lungs. In addition, enrichment of the mTORC1 signalling gene set in transcriptomic data from patients with fibrotic lungs in the large lung study from the GRADS investigators strengthened the importance of this pathway in fibrotic sarcoidosis.

The high level of T-lymphocytes and accumulation of Th17/Th17.1 cells in the lungs implicates aberrant regulatory elements for example loss of control of T-cell proliferation by immune cells with regulatory function *e.g.* iNKT cells [137] and Tregs [4], which were deficient and abnormal respectively in the lungs of sarcoidosis patients. However, none of these studies investigated these changes in fibrotic sarcoidosis. In addition, many of the works cited (other than the GRADS study) were on small numbers of patients.

Much of this piecing together of information also involved the use of data from progressive *versus* nonprogressive patients, where we argued that this was a minimal requirement for fibrosis. However, this is not proven and it is unclear if this is a *sine qua non* for fibrosis. It is also not clear which of these pathways are critical and how many of these factors have to co-exist to result in progressive fibrotic disease in sarcoidosis. Of course, one reason for the gap in understanding is the lack of a robust animal model for pulmonary sarcoidosis (let alone fibrotic sarcoidosis). Although models for granulomatous inflammation exist, *e.g. Cutibacterium acnes*, mycobacterial catalase–peroxidase and ESAT-6 models, leprosy, and schistosomiasis [138], it is probably not a model of the complex disease itself and certainly not of fibrotic sarcoidosis. It is debatable whether a model for a complex disease like sarcoidosis could really ever provide translatable information and more studies comparing fibrotic *versus* nonfibrotic human disease are likely to be more informative. Lung tissue studies, ideally matched to blood analysis, are particularly needed. Such studies could also yield information on a biomarker to identify patients who are more at risk of fibrosis. Potential blood biomarkers include serum IL-17 levels, monocyte levels and CCL-18 in BAL samples.

We conclude that there is enough evidence to suggest the importance of T-lymphocytes and chronic mTORC1 signalling in fibrotic sarcoidosis. We have avoided a didactic conclusion as most of the work does not involve a comparison of fibrotic *versus* nonfibrotic sarcoidosis; many had fewer than 10 fibrotic sarcoidosis patients and there are negligible murine studies of fibrotic granulomatous processes. With the data that are currently available, our perspective is that chronic fibrosis in sarcoidosis is likely to be driven by a combination of increased Th17 cells, possibly due to resistance to apoptosis, combined with primed monocyte-derived macrophages (*TLR3* polymorphism, type 1 IFN signalling) which may respond disproportionately to infection (acute, low grade or the microbiome) (summarised in table 2). This drives the production of CCL-18 from macrophages, which further attracts activated CD4 T-cells and increases TGF-β secretion from surrounding structural and immune cells. An abnormal Treg potentially enhances the Th17 numbers and the persistence of a specific type of antigen may also contribute to chronic Th17-mediated inflammation. The strongest data are around the mTORC1 upregulation signal, where there is also pathobiology to support this as a mechanism for persistence of granuloma. This upregulation is, however, likely to be downstream of a large number of intra- and extracellular immune and metabolic signals.

**TABLE 2:** Key immune abnormalities linked to references, and whether these are found in patients with fibrotic *versus* nonfibrotic sarcoidosis; those in progressive compared to nonprogressive or resolved sarcoidosis; or if they were only compared between sarcoidosis patients with abnormal and normal lung function tests

**Immune abnormalities**	**Reference**	**Fibrotic *versus* non-fibrotic sarcoidosis**	**Progressive *versus* nonprogressive or resolved**	**Abnormal *versus* normal lung function**	**Blood**	**Lung/BAL**	**Key message**
***TGFB*** **polymorphisms**	[24, 25]	Yes	Yes	No evidence	No evidence	No evidence	Association with fibrotic sarcoidosis
***GREM1* polymorphisms**	[31]	Yes	No evidence	No evidence	No evidence	No evidence	Association with fibrotic sarcoidosis
***TLR2* polymorphisms**	[36, 37]	No evidence	Yes	No evidence	No evidence	No evidence	Association with chronic progressive sarcoidosis
***TLR3* polymorphisms**	[38]	No evidence	Yes	Yes	No evidence	No evidence	Association with chronic progressive sarcoidosis
***ANXA11* polymorphisms**	[44]	Yes	No evidence	No evidence	No evidence	No evidence	Association with chronic progressive sarcoidosis
**Overall transcriptome of lung tissue**	[47]	Yes	Yes	No evidence	No evidence	Yes	Activated lymphocytes linked to progressive/fibrosis
**Transcriptome of blood cells**	[48]	Yes	Yes	Yes	Yes	Yes	Type 1 IFN linked to abnormal lung function, type 1 and 2 IFNs to progressive/fibrosis
**Bulk RNA-seq of BAL**	[54]	Yes	Yes	No evidence	No evidence	Yes	mTORC1 signalling linked to fibrosis
**SAA**	[63, 64]	Yes	No evidence	Yes	Yes	Yes	SAA linked to collagen levels in lung tissue and higher in serum of fibrotic sarcoidosis
**CCL-18**	[72, 73]	Yes	Yes	No evidence	No evidence	Yes	CCL-18 levels in BAL increase with increase in Scadding stages
**TGF-β**	[26, 27]	Yes	No evidence	Yes	No evidence	Yes	Higher levels in BAL of abnormal lung function [26] but lower in fibrotic CXRs [27]
**CCL-17**	[73]	Yes	No evidence	No evidence	No evidence	Yes	CCL-17 levels in BAL increase with increase in Scadding stages
**Monocytes**	[76]	No evidence	Yes	No evidence	Yes	Yes	Higher intermediate monocytes in blood and TNF-α-producing monocytes in BAL of chronic progressive sarcoidosis
**CD163^+^ macrophage**	[80]	No evidence	Yes	No evidence	No evidence	Yes	Higher levels of CD163^+^ macrophages in lung sections in those with progressive disease; too few patients to call for those with fibrosis
**Th17/17.1**	[3]	No evidence	Yes	No evidence	Yes	Yes	Th17.1 and BAL IL-17 levels higher in progressive disease
**PD-1^+^ CD4 T-cells**	[101]	No evidence	Yes	No evidence	Yes	No evidence	PD-1^+^ CD4 T-cells higher in progressive disease

Blood and lung/BAL indicate if these findings were in blood or lungs. Caveat to the strength of evidence including number of patients is expanded in text. BAL: bronchoalveolar lavage; CCL: C-C motif chemokine ligand; CXR: chest radiograph; IFN: interferon; mTORC1: mammalian target of rapamycin complex 1; PD: programmed cell death; RNA-seq: RNA sequencing; SAA: serum amyloid antigen; TGF-β: transforming growth factor-β; Th: T-helper; TLR: Toll-like receptor.

A better understanding of these mechanisms in fibrotic sarcoidosis has become a lot more relevant with the introduction of nintedanib as an antifibrotic for progressive fibrotic sarcoidosis. Nintedanib is a small molecule selective tyrosine kinase receptor inhibitor that targets vascular endothelial growth factor, fibroblast growth factor and platelet-derived growth factor receptors. However, it also inhibits nonreceptor tyrosine kinases (Src, Lck and Lyn families) involved in wide-ranging cellular processes such as proliferation, differentiation and adhesion [139]. The Lck family of tyrosine kinases may be most relevant here due to its requirement for T-cell activation and survival [140, 141]. It is therefore theoretically possible that nintedanib could be particularly useful for fibrotic sarcoidosis as it would target both the fibrotic tyrosine kinase receptor inhibitors and the T-cell tyrosine kinases. As proposed, the latter could “hit” the Th17 T-cells proposed as a key driver of fibrogenesis in sarcoidosis. However, it could also reduce the activity of Tregs further, and is narrower in repertoire compared to corticosteroids as an immunosuppressant and anti-inflammatory. The relative contribution of monocytes and macrophages, for example, is likely to be significant and may not be affected by nintedanib. At this point, there is not enough clinical data yet to support its lone use in fibrotic sarcoidosis and we propose that established immunosuppressants are used first in fibrotic sarcoidosis patients and, at least, used with nintedanib for a period of time during the introduction of nintedanib treatment. Our review raises the possibility of repurposing other immune modulating drugs; for example, those that target the IL-17 and mTORC signalling pathways. However, we end with an oft-used but particularly relevant statement that more research in fibrotic sarcoidosis is greatly needed; not only searches for drugs but also better designed clinical trials for this group of patients.

**Points for clinical practice**

- The disease mechanism in fibrotic pulmonary sarcoidosis is poorly understood and under researched.
- There are very few studies that directly compare the immune profile between patients with fibrotic sarcoidosis and those who did not progress to fibrosis. The few studies that did this highlighted the importance of T-lymphocytes and chronic mTORC1 signalling in fibrotic pulmonary sarcoidosis.
- A better understanding of these mechanisms in fibrotic sarcoidosis has become a lot more relevant with the introduction of nintedanib as an antifibrotic for progressive fibrotic sarcoidosis.
- There is mechanistic evidence for the use of nintedanib in fibrotic pulmonary sarcoidosis but there is not enough clinical data yet to support its lone use in fibrotic sarcoidosis.
- We propose that established immunosuppressants are used first in fibrotic sarcoidosis patients or at least used with nintedanib for a period of time during the introduction of nintedanib treatment.
